# Use of a chemically induced-colon carcinogenesis-prone *Apc*-mutant rat in a chemotherapeutic bioassay

**DOI:** 10.1186/1471-2407-12-448

**Published:** 2012-10-03

**Authors:** Kazuto Yoshimi, Takao Hashimoto, Yusuke Niwa, Kazuya Hata, Tadao Serikawa, Takuji Tanaka, Takashi Kuramoto

**Affiliations:** 1 Institute of Laboratory Animals, Graduate School of Medicine, Kyoto University, Yoshidakonoe-cho, Sakyo-ku, Kyoto 606-8501, Japan; 2 Sunplanet Co., Ltd, 4388 Makita, Kamiishizu, Ogaki, 503-1602, Japan; 3 Cancer Research and Prevention, The Tohkai Cytopathology Institute, 4-33 Minami-Uzura, Gifu, 500-8285, Japan

**Keywords:** Adenomatous polyposis coli, Colorectal cancer, Endoscopy, Rat, Chemotherapy, 5-fluorouracil

## Abstract

**Background:**

Chemotherapeutic bioassay for colorectal cancer (CRC) with a rat model bearing chemically-induced CRCs plays an important role in the development of new anti-tumor drugs and regimens. Although several protocols to induce CRCs have been developed, the incidence and number of CRCs are not much enough for the efficient bioassay. Recently, we established the very efficient system to induce CRCs with a chemically induced-colon carcinogenesis-prone *Apc*-mutant rat, Kyoto Apc Delta (KAD) rat. Here, we applied the KAD rat to the chemotherapeutic bioassay for CRC and showed the utility of the KAD rat.

**Methods:**

The KAD rat has been developed by the ENU mutagenesis and carries a homozygous nonsense mutation in the *Apc* gene (S2523X). Male KAD rats were given a single subcutaneous injection of AOM (20 mg/kg body weight) at 5 weeks of age. Starting at 1 week after the AOM injection, they were given 2% DSS in drinking water for 7 days. Tumor-bearing KAD rats were divided into experimental and control groups on the basis of the number of tumors observed by endoscopy at week 8. The 5-fluorouracil (5-FU) was administrated intravenously a dose of 50 or 75 mg/kg weekly at week 9, 10, and 11. After one-week interval, the 5-FU was given again at week 13, 14, and 15. At week 16, animals were sacrificed and tumor number and volume were measured macroscopically and microscopically.

**Results:**

In total 48 tumors were observed in 27 KAD rats with a 100% incidence at week 8. The maximum tolerated dose for the KAD rat was 50 mg/kg of 5-FU. Macroscopically, the number or volume of tumors in the 5-FU treated rats was not significantly different from the control. Microscopically, the number of adenocarcinoma in the 5-FU treated rats was not significantly different (p < 0.02) from that of the control. However, the volume of adenocarcinomas was significantly lower than in the control. Anticancer effect of the 5-FU could be obtained only after the 16 weeks of experimental period.

**Conclusion:**

The use of the AOM/DSS-treated tumor-bearing KAD rats could shorten the experimental period and reduce the number of animals examined in the chemotherapeutic bioassay. The efficient bioassay with the AOM/DSS-treated tumor-bearing KAD rats would promote the development of new anti-tumor drugs and regimens.

## Background

Chemotherapeutic bioassays for colorectal cancer (CRC) play an important role in the development of new anti-tumor drugs and regimens. These bioassays involve the use of colon carcinogenesis models which mainly consist of animal xenografts, an adenomatous polyposis coli (*Apc*)-mutant mouse model and a chemically-induced CRC model [1-3].

The xenograft model utilizes cultured or primary CRC cells that are implanted under the skin of immune-deficient mice and rats. The size and volume of tumors can be estimated easily and temporally by measuring their dimensions. However, these animals have defects in the immune system that suppresses tumor growth. The subcutaneous microenvironment around the transplanted tumors differs from the colon environment in which the original CRC of the cell lines arose. Therefore, this approach appears to ignore the contribution of the tumor microenvironment and does not exactly mimic tumor development in man [4,5].

*Apc*-mutant mouse models, such as the Min mouse model, spontaneously develop a considerable number of intestinal tumors and have been widely used as a relevant model for evaluating human chemopreventative therapies. However, tumors in the colon are developed at a much lower frequency than in the small intestine. Even if tumors do develop in the colon, almost all of them are low grade adenomas [6].

The chemically-induced CRC model is superior to these models in that the characteristics of the induced tumor are very similar to those of human CRC. Tumors only develop in the colon through multi-step carcinogenesis which mimics the entire process of tumor growth in man. In this model, tumor morphology and mutation spectrum are also similar to those in human CRC [6]. Moreover, methods of inducing colon tumors are well-established, so that we can be certain of obtaining the number of tumors expected, which is ideal for the evaluation of potential chemotherapeutic drugs [2,7].

Although many carcinogens induce colon tumors in rats, azoxymethane (AOM) administered subcutaneously has been most widely used [2,6,8]. However, the incidence of colon tumors induced by two or three subcutaneous injections of AOM is not high, and it takes 7–9 months to induce sufficient tumors to evaluate the chemotherapeutic efficacy of potential anti-cancer drugs [9]. Such limitations have been significantly improved by using dextran sodium sulfate (DSS) as an inflammatory agent. When 2% DSS is administered in drinking water to the AOM-treated rats for one week, starting one week after administration, a number of colon tumors develop within a short time period (this is known as the TANAKA method) [10].

Recently we developed a novel *Apc* mutant rat strain, called the Kyoto Apc Delta (KAD) rat (strain name: F344-*Apc*^*m1kyo*^) from our ENU-mutagenesis program. The KAD rat carries a homozygous nonsense mutation in the *Apc* gene (S2523X). Thus, the KAD rat lacks 321-amino acids in the C-terminal of APC, but it remains viable at almost 2 years and shows no spontaneous colorectal tumors. Moreover, by applying the TANAKA method to KAD rats, we obtained a much higher incidence, multiplicity and malignancy of colon tumors in KAD rats than colon tumors in F344 wild rats. We were able to induce these tumors within 15 weeks of the experimental period. In addition, we were able to carry out endoscopic observation, by which colon tumors could be detected from Week 8 [11].

In the present study, in order to establish an efficient chemotherapeutic bioassay with KAD rats, we induced colon tumors by means of treatment with AOM and DSS, and then administered a typical anti-tumor drug, namely 5-fluorouracil (5-FU) to the tumor-bearing rats.

## Methods

### Chemicals

5-FU was purchased from Kyowa Hakko Kogyo, Co., Ltd. (Tokyo, Japan). AOM was purchased from Sigma-Aldrich Chemical Co. (St. Louis, MO, USA). These drugs were diluted in saline just before administration. DSS (MW 36,000–50,000) was purchased from ICN Biochemicals, Inc. (Aurora, OH, USA). DSS was dissolved in distilled water at 2% (w/v) every day before treatment.

### Rats

Specific pathogen free male KAD rats were purchased from Japan SLC, Inc. (Hamamatsu, Japan) and provided by the National Bio Resource Project for the Rat (http://www.anim.med.kyoto-u.ac.jp/nbr) at 4 weeks of age. The rats were acclimatized for a week before the experiment and were maintained under conditions of 50 ± 10% humidity, 12 h-12 h light cycle and 24 ± 2 °C temperature. They were fed a standard pellet diet (F-2, Funabashi Farm, Funabashi, Japan) and tap water *ad libitum*.

### Induction of colon tumor

**Figure 1:**
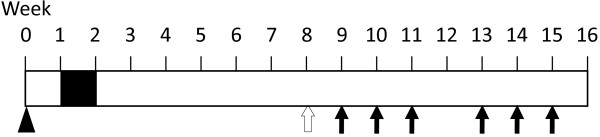
**Experimental schedule.** KAD rats at 5 weeks of age were given a subcutaneous injection of AOM at 20 mg/kg body weight (arrow head). One week after the AOM injection they were given 2% DSS (MW 36,000–50,000) in their drinking water for one week (black box). Endoscopic observation was carried out at week 8 (open arrow). 5-FU was administrated intravenously at weeks 9, 10, 11, 13, 14 and 15 (arrows). All animals were sacrificed at week 16.

Chemically induced-colon carcinogenesis was carried out as described in our previous study [11]. Briefly, male KAD rats (n = 32) were given a single subcutaneous injection of AOM (20 mg/kg body weight) at 5 weeks of age. Starting at 1 week after the AOM injection, they were given 2% DSS in drinking water for 7 days (Figure 1). Five rats were used to find correlation of the number of polypoid lesions with the volume of tumors at Week 8. All experimental procedures were approved by the Animal Research Committee of Kyoto University and were performed according to the Regulation on Animal Experimentation at Kyoto University.

### Endoscopic observation

Observation was performed at week 8 with an endoscope (BF TYPE 3C40: Olympus, Tokyo, Japan) to determine the presence of colon tumors (Figure 1). KAD rats were anesthetized by administration of 2% isoflurane (Forane: Abbott Japan, Tokyo, Japan) vapor through a nose cone. The colon was flushed using an enema of tap water to remove feces. The endoscope was inserted into the colon, and endoscopic images were acquired within the distal colon and rectum. The numbers of polypoid lesions, assumed to be developing colorectal tumors, were counted.

### Chemotherapeutic test

The AOM/DSS-treated rats were divided into three groups (nine rats each), among which numbers of colon tumors were not significantly different. The 5-FU was administrated to the tumor-bearing KAD rats at two different doses (50 or 75 mg/kg) by three weekly intravenous (i.v.) injections at weeks 9, 10 and 11. According to the preliminary experiment, we set a 1 week withdrawal period to decrease the occurrence of serious side effects caused by 5-FU. One week later, rats underwent additional administration of 5-FU involving three weekly i.v. injections at weeks 13, 14 and 15. At week 16 animals were sacrificed by cervical dislocation under anesthesia with isoflurane (Figure 1). Then the colorectum of the rats was resected, washed with PBS, opened longitudinally along the main axis and fixed in 10% neutral buffered formalin for at least 24 h. The number and volume of colon tumors were measured after fixation. The other organs such as small intestine, stomach, liver and kidney were observed macroscopically for any abnormalities.

### Histopathological examination

After careful macroscopic inspection, tumors and whole colonic mucosa were embedded in paraffin and sectioned for histopathology after staining with hematoxylin and eosin. After tumors that developed in the colorectum were photographed, the largest and the smallest superficial diameters of adenocarcinoma that were diagnosed histopathologically were measured on the photographs. Tumor volume was calculated according to the formula V = a × b^2^/2, in which “a” is the largest superficial diameter and “b” is the smallest superficial diameter [12].

### Immunohistochemistry

Cell proliferation and apoptosis were evaluated by determination of the percentages of PCNA- and cleaved caspase-3-positive nuclei in a total of 200 cancer cells for each sample (n = 6 from the control group and n = 8 from Group 1). Briefly, sections were incubated with anti-mouse PCNA antibody (clone PC10, 1:1000 dilution; DAKO) and cleaved caspase-3 (Asp175) antibody (1:1000 dilution; Cell Signaling Technology) overnight at 4 °C. Biotinyl antibody was used as secondary antibody and then the streptavidin-peroxidase complex (LASB^TM^' + Kit, Universal, DAKO) was applied. The antigen-antibody complex was visualized by 3,3′-diamonobenzidine tetrachloride (DAKO).

### Statistical analysis

Data are expressed as the mean ± standard deviation (S.D.). Student’s *t*-test was performed using the statistics package within Microsoft Excel for statistical analysis, and p values were considered significant when < 0.05.

## Results

### Correlation of the number of polypoid lesions with the total volume of tumors

To find the correlation of the number of polypoid lesions with the total volume of tumors, we induced colon tumors to KAD rats (n = 5) by the TANAKA method and counted tumor number under the endoscopy and the number and volume of tumors under the microscopy at week 8 (Additional file 1: Table S1). As a good correlation between them was found, it is very likely that the number of polypoid lesions found with the endoscopy at week 8 can be used to estimate the total volume of tumors (Additional file 2: Figure S1).

### Effective tumor development in AOM/DSS-treated KAD rats

At week 8 when carrying out endoscopic observations for the occurrence of colon tumors in the colons of AOM/DSS-treated KAD rats, we could observe about 10 cm of the luminal surface, from the rectum to the distal colon. We found polypoid lesions around the rectum and the distal colon. Polypoid lesions which were clearly different from normal mucosa assumed to be developing colorectal tumors. All AOM/DSS-treated KAD rats developed colon tumors. In total 48 tumors were observed in 27 KAD rats with a 100% incidence and a multiplicity of 1.78 ± 0.85, ranging from 1 to 4 per rat.

### Dosing condition of 5-FU

**Figure 2:**
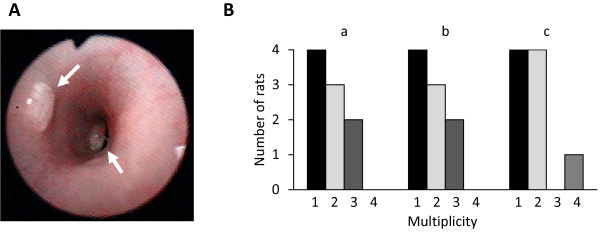
**Grouping of AOM/DSS-treated KAD rats before 5-FU treatment.** (**A**) Endoscopic view of colon tumors (arrows) in an AOM/DSS-treated KAD rat at week 8. (**B**) AOM/DSS-treated KAD rats were divided into experimental groups based on the number of tumors induced in their colons. The number of tumors induced in each animal determined by endoscopic observations varied from one to four. In total, 48 tumors were found in 27 rats. The tumor-bearing rats (nine per group) were divided into three groups (a: saline, b: 50 mg/kg 5-FU and c: 75 mg/kg 5-FU), so as not to be significantly different at the starting point of treatments.

On the basis of the number of tumors, the tumor-bearing KAD rats were divided into three groups. One was the control group and the others were experimental groups, in which rats were given 5-FU at a concentration of 50 mg/kg (Group 1) or 75 mg/kg (Group 2). Each group consisted of nine rats and the total number of tumors in each group was 16. The average number of tumors per rat was not significantly different among the groups (Control: 1.78 ± 0.83, Group 1: 1.78 ± 0.83, Group 2: 1.78 ± 0.97) (Figure 2B).

**Figure 3:**
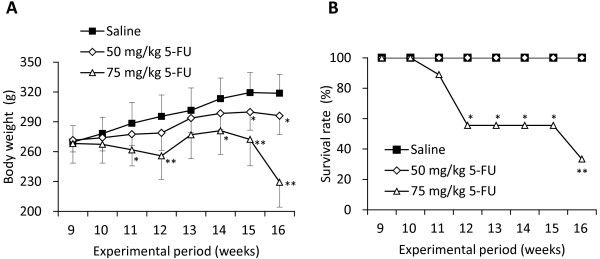
**Toxicity of 5-FU in AOM/DSS treated KAD rats.** Average body weight (**A**) and survival rate (**B**) of the control, 50 mg/kg and 75 mg/kg 5-FU treated groups. *p < 0.05, **p < 0.001.

The average body weight of rats in Group 1 tended to be lower than in the control group, and was significantly different from the control group at weeks 15 (300.0 ± 18.1 vs 319.4 ± 20.1; p < 0.05) and 16 (296.1 ± 18.9 vs 318.8 ± 18.8; p < 0.03). However the reduction in body weight was less than 10% as compared with the control. None of the rats in Group 1 died during the experiment. On the other hand, gain in body weight in Group 2 was constantly and significantly impaired throughout the experimental period. More than 10% of weight loss was observed at weeks 12, 15 and 16, as compared with that in the control group (Figure 3A). KAD rats in Group 2 had severe bloody stools and diarrhea and six rats (67%) in the group died during the experiment (Figure 3B). These findings indicated that the 75 mg/kg dose of 5-FU was too toxic for the tumor-bearing KAD rats, and led to the marked body weight loss and eventually to death. Thus, the 50 mg/kg dose of 5-FU was considered to be appropriate for evaluation of the antitumor activity of 5-FU, when we used the tumor-bearing KAD rats in a chemotherapeutic bioassay.

### Reduction of volume but not number of adenocarcinomas in the tumor-bearing KAD rats by treatment with 5-FU

**Figure 4:**
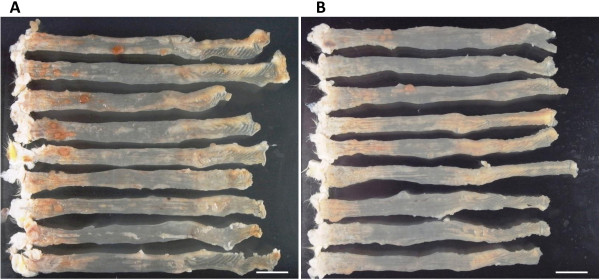
**Macroscopic view of large bowel.** Macroscopic view of large bowel of KAD rats that were not given 5-FU (**A**) and that were given 50 mg/kg 5-FU (**B**). Colon tumors that developed in both groups were mainly distributed in the rectum and distal colon, which was assumed to be within 8 cm from the anus. No tumors were observed in the proximal colon. Left side: anus. Right side: cecum. Bar: 2 cm.

At week 16 we carried out an autopsy and macroscopic examination of the large bowels of the control group and Group 1. Macroscopically, rats in both groups developed multiple nodular, polypoid or caterpillar-like tumors mainly in the rectum and distal colon (Figure 4). The number of tumors in Group 1 was not significantly different from the control group (Table 1). The volume of tumors, which were macroscopically calculated, was 27% smaller in Group 1 than in the control group, but the difference was not significant (p = 0.34).

**Table 1:** Effects of 5-FU on the development of colon tumors in the KAD rat

**Treatment**	**No. of rats**	**Macroscopic observation**		**Microscopic observation**			
		**Multiplicity**	**Volume (mm**^**3**^**)**^**1**^	**Multiplicity**			**Volume (mm**^**3**^**)**^**2**^
				**adenomas**	**adenocarcinomas**	**total**	**adenocarcinoma**
Saline	9	5.56 ± 3.43	106.34 ± 68.92	3.11 ± 2.52	3.22 ± 2.77	6.33 ± 4.87	63.85 ± 51.06
50 mg/kg 5-FU	9	6.33 ± 3.04	77.28 ± 57.23	3.78 ± 1.79	2.33 ± 1.94	6.11 ± 2.37	34.40 ± 31.26 ^3^

^1^Tumor volume was determined by the formula V = a × b^2^/2 (V: volume. a: the largest superficial diameter and b: the smallest superficial diameter).

^2^Volumes of adenomas were too small to calculate.

^3^Adenocarcinoma volumes observed in the 5-FU-treated KAD rats were significantly reduced as compared with those of non-treated rats (p <0.02).

**Figure 5:**
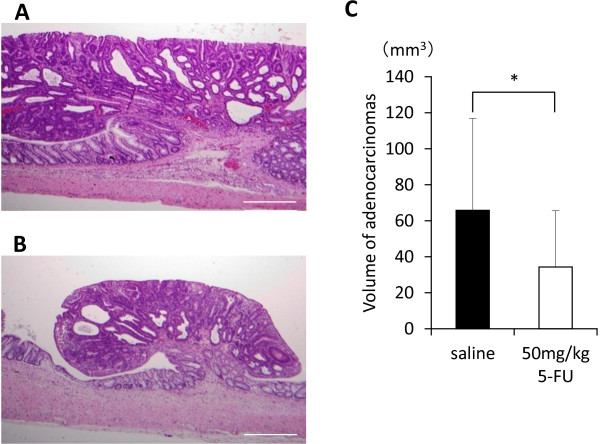
**Histopathological analysis of colonic adenocarcinomas.** Colonic adenocarcinoma developed in KAD rats that were not given 5-FU (**A**) and that were given 50 mg/kg 5-FU (**B**). Most of the adenocarcinomas in KAD rats that received 50 mg/kg 5-FU were smaller than those in the control group (hematoxylin and eosin stain). Bar: 500 μm. (**C**) Average volume of adenocarcinoma tumors grown in KAD rats that were given saline or 5-FU (50 mg/kg). The mean volume of adenocarcinoma tumors in the 5-FU-treated rats were significantly smaller than those in the non-treated rats. *: p < 0.02.

**Figure 6:**
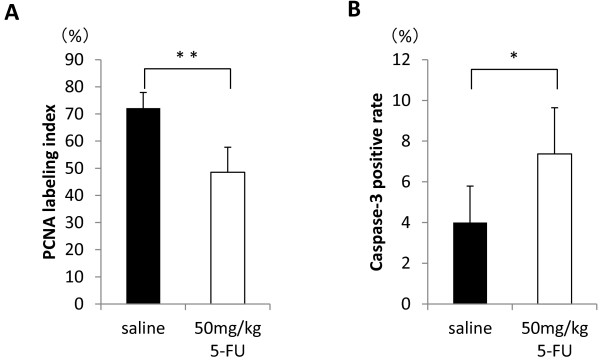
**Cell proliferation and apoptosis in adenocarcinomas of the 5-FU treated KAD rats.** (**A**) The PCNA labeling index was significantly lower in the 5-FU treated KAD rats (50 mg/kg) than that in the control KAD rats, indicating significant decrease in cell proliferation in the adenocarcinomas. **: p < 0.001. (**B**) The cleaved caspase-3 positive rate was significantly higher in the 5-FU treated KAD rats (50 mg/kg) than that in the control KAD rats, indicating significant increase in apoptosis in the adenocarcinomas. *: p < 0.02.

Microscopically, all tumors that developed in KAD rats were tubular adenoma or well- or moderately-differentiated tubular adenocarcinoma (Figure 5A and 5B). The multiplicity of adenoma or adenocarcinoma in Group 1 was not significantly different from that of the control group (p = 0.53; p = 0.44, respectively) (Table 1). The size of the adenomas was too small for their volumes to be calculated. However, the volume of adenocarcinomas (63.85 ± 51.06 mm^3^) in Group 1 was significantly lower (p < 0.02) than in the control group (34.40 ± 31.26 mm^3^), when the volume was calculated from the histological sections. In addition, we found significant reduction of PCNA labeling index as well as significant elevation of cleaved caspase-3 positive rate in the adenocarcinomas in Group 1 (Figure 6). These results suggest that the 5-FU treatment suppressed cell proliferation and induced apoptosis and thereby inhibited adenocarcinoma development.

## Discussion

Carcinogenic process is complex. Tumor development proceeds via a multi-step process, in which a succession of genetic changes, each conferring one or another type of growth advantage, leads to the progressive conversion of normal cells into cancer cells. Moreover, extent of cell transformation depends on the genetic predisposition and environmental factors [13]. Thus, to obtain cancerous lesions effectively, it is necessary to use a synergy effect of genetic and environmental factors. Our carcinogenic system with KAD rats employs such synergy effect of *Apc*-mutation, chemical carcinogen exposure, and tissue inflammation.

In ideal chemotherapeutic bioassay systems, the number and volume of tumors should be evaluated as the indicator of anti-tumor drug efficacy. Therefore, it is indispensable to be able to strictly set the size of the experimental and control groups, among which the number and volume of tumors should not differ significantly. To this end, we carried out endoscopic observations in the colons of AOM/DSS-treated KAD rats, and divided animals into groups on the basis of the number of colon tumors. Since the rat has a suitable body size for handling, we could easily manipulate the endoscope and correctly count the number of tumors. At week 8 we found colon tumors with a 100% incidence in AOM/DSS-treated KAD rats. The rats developed one to four tumors. On the basis of the number of tumors, we could set the experimental and control groups, because the number of tumors observed by the endoscopy is correlated to the volume of tumors obtained by the microscopy at week 8. We, therefore, recommend counting the number of tumors using endoscopic observation before dividing the rats into groups.

It is important to identify biomarkers that are used to predict efficacy and safety of anti-tumor drugs. Rats can be subjected to the sequential sampling of bloods. The amounts of bloods or urines are enough to be examined. Moreover, drug kinetics can be monitored by *in vivo* imaging [14]. Thus, the chemotherapeutic bioassay with the KAD rats is a candidate system to explore the biomarkers.

5-FU is a pyrimidine analog and when incorporated into DNA inhibits the cell’s ability to synthesize DNA. Eventually 5-FU induces cell cycle arrest and apoptosis, mainly in cells with high proliferative activity such as cancer cells [15]. Side effects of 5-FU, such as diarrhea and weight loss, are problematic in performing chemotherapeutic tests with animal models. Thus, it is important to determine the maximum tolerated dose (MTD) that does not produce profound weight loss, and that causes no drug-related lethality. Usually the MTD of 5-FU in rats ranges from 25 to 100 mg/kg, depending on the 5-FU administration schedules [16]. In the current study, we found that the MTD was 50 mg/kg of 5-FU when administered to tumor-bearing KAD rats by i.v. injection. Although the MTD should be determined using different administration schedules and routes, the MTD that we determined in the present study can be a helpful guide in setting doses of anti-cancer drugs in further chemotherapeutic tests with KAD rats.

In our study, the treatment of tumor-bearing KAD rats with 5-FU failed to reduce the multiplicity of adenoma or adenocarcinoma. However, the treatment significantly reduced adenocarcinoma tumor volume and cell proliferation as well as increased adenocarcinoma apoptosis, which was consistent with the mode of action of the 5-FU [15]. Treatment response assessed in terms of change in tumor size after 5-FU administration in the present study amounted to a 30% reduction, which was similar to the response rate of 5-FU as a single agent seen in human cancers, including CRC [17]. These findings indicated that the response of tumors in AOM/DSS-treated KAD rats to 5-FU treatment was similar to human CRC, and supported the view that this should be a useful bioassay system for employment in further chemotherapeutic studies.

## Conclusions

In the present study we established a chemotherapeutic bioassay system for CRC using KAD rats. In this system, we could set the experimental groups on the basis of the number of tumors detected by endoscopic examination. After 5-FU administration rat colon tumors induced by AOM/DSS treatments showed a similar response, in terms of percentage reduction in size and cell proliferation and percentage elevation in apoptosis, to those reported in clinical CRC studies. Thus, we expect that this system could effectively promote the development of new anti-tumor drugs and regimens for human CRC.

## Competing interests

The authors declare that they have no competing interests.

## Authors’ contributions

KY and TK conceived the study and designed the experiments. KY, TH, YN and KH performed the experiments. TT performed the histopathological analysis. KY, TK and TT wrote the manuscript. TS revised the manuscript. All authors have read and approved the final manuscript.

## Pre-publication history

The pre-publication history for this paper can be accessed here:

http://www.biomedcentral.com/1471-2407/12/448/prepub

## Supplementary Material

Additional file 1 Table S1. Number and total volume of tumors found in KAD rats at week 8. Click here for file

Additional file 2 **Figure S1. The correlation of total volume of tumors with the numbers of polypoid lesions observed by endoscopy at Week8.** Regression formula was made with Excel software package (Microsoft). Vertical axis was shown in logarithmic scale. Click here for file
