# Supplementary material for: Use of a chemically induced-colon carcinogenesis-prone Apc-mutant rat in a chemotherapeutic bioassay
Source: BMC Cancer. 2012 Oct 3;12:448. doi: 10.1186/1471-2407-12-448 (PMC3517449; doi:10.1186/1471-2407-12-448)
Supplement: Additional file 1 — Table S1. Number and total volume of tumors found in KAD rats at week 8. [file 1471-2407-12-448-S1.doc]

# Supplemental data

## Supplementary Table 1. Number and total volume of tumors found in KAD rats at Week 8

| Rat ^1^ | Number of polypoid lesions observed by endoscopy | Number of adenoma found in the histopatholgocial examination | Number of dysplasia found in the histopatholgocial examination | Total volume of tumors (mm^3^) ^2^ |
| --- | --- | --- | --- | --- |
| 1 | 0 | 0 | 1 | 0.90 |
| 2 | 1 | 0 | 1 | 3.49 |
| 3 | 1 | 0 | 2 | 1.76 |
| 4 | 6 | 3 | 3 | 2736.5 |
| 5 | 0 | 0 | 0 | 0.00 |

1; Five AOM/DSS-treated male KAD rats were used.
2; Total volume of tumors were correlated with the number of polypoid lesions observed by endoscopy at Week 8 (R^2^ = 0.99) (see Supplementary Figure 1).
