# Supplementary figures and images for: Use of a chemically induced-colon carcinogenesis-prone Apc-mutant rat in a chemotherapeutic bioassay
Source: BMC Cancer. 2012 Oct 3;12:448. doi: 10.1186/1471-2407-12-448 (PMC3517449; doi:10.1186/1471-2407-12-448)

## Slide 1
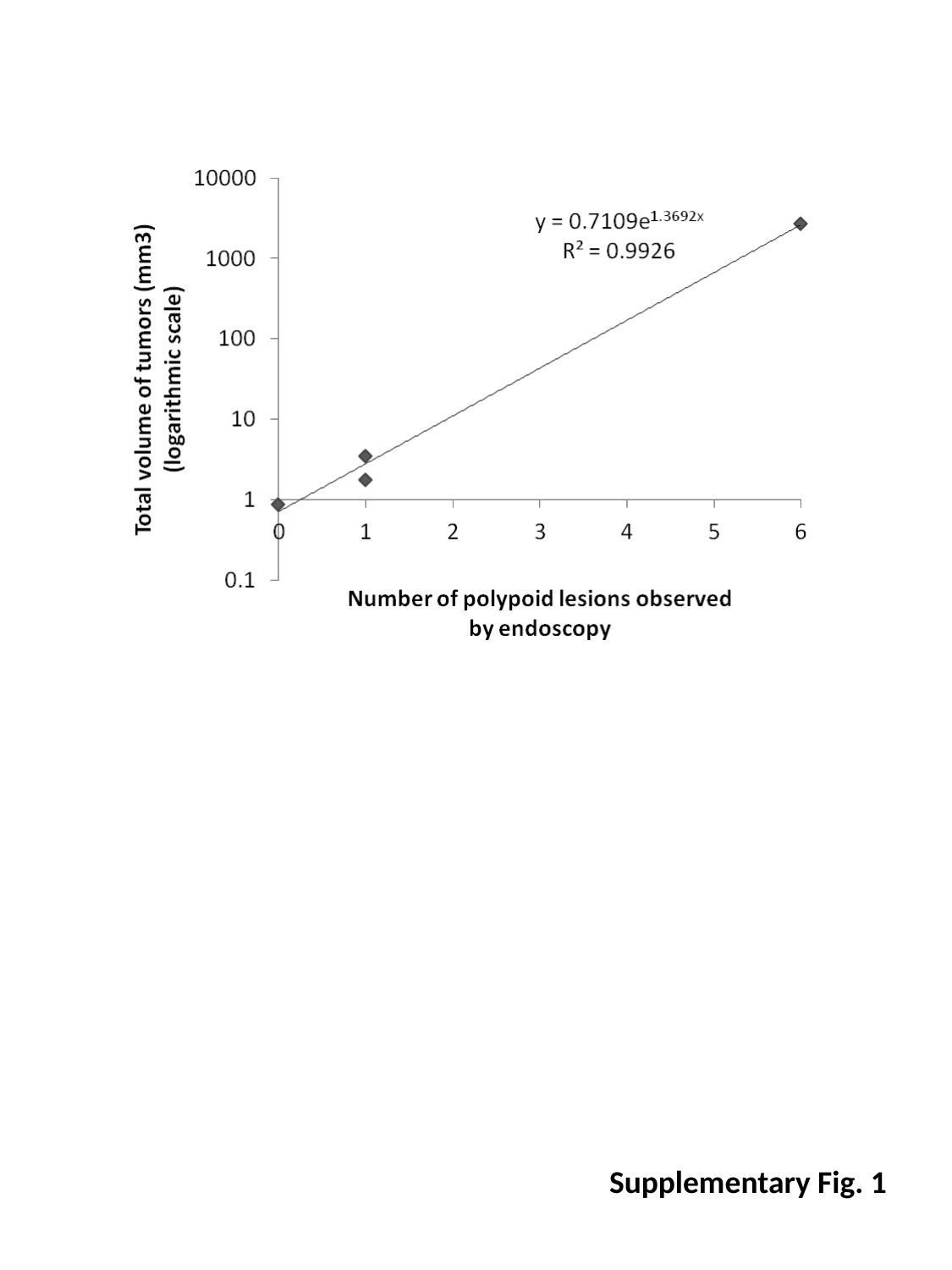

Supplementary Fig. 1

Supplement: Additional file 2 — Figure S1. The correlation of total volume of tumors with the numbers of polypoid lesions observed by endoscopy at Week8. Regression formula was made with Excel software package (Microsoft). Vertical axis was shown in logarithmic scale. [file 1471-2407-12-448-S2.ppt]
